# Gleanings from Our Exchanges

**Published:** 1874-01-15

**Authors:** 


THE VARIOLA-VARICELLA QUESTION.—Kaposi (*Archiv fur Dermat. und Syph.*, V. Jahr. Zweites Heft), in a long and important article, in which this much-vexed question is considered, with particular reference to the many points which have been put forward by those who maintain the duality or individuality of the two affections, draws, from all the premises, the conclusion that varicella, as a contagious affection, is identical with variola. In the same number will be found a report in full of a discussion of this question, which occupied several sessions of the Vienna Medical Society.—*Boston Med. and Surg. Jour.*

MENSTRUATION AND OVULATION.—The entirely separate character of these two physiological processes is argued by Dr. H. Beigel, in the *Weiner Medicinische Wochenschrift*. Further, conception is independent of menstruation. Young women conceive before they menstruate; in cases of double ovariotomy, women menstruate when they cannot conceive. Menstruation he defines as "a periodically recurrent sexual impulse," the exact signification of which expression is obscure to us.—*Philadelphia Medical Reporter*.

A Case of Poisoning by Five Grains of Strychnine Treated by Chloroform Inhalations. — Recovery.—As the following case may be of some interest, I will submit it to the profession:

Mr. B., shop-keeper, a middle-aged man of temperate habits, while suffering from depression of spirits, obtained, on September 1st, ten grains of strychnine, representing his intentions were to poison a dog. He secured a room at a hotel, took a dose of laudanum as a preparatory step, and went to bed, intending to swallow the drug as soon as the effects of the opiate were apparent. It appears he fell asleep, and did not awake till half-past four in the morning, when he took half of the quantity previously mentioned. Some time after, he was seized with convulsions. The occupants of the adjoining rooms, awakened and alarmed by his screams, at length came to his relief. I was called, and saw him at 6 A.M. I found him lying in bed; legs and arms extended, his hands firmly clenching the sides of the mattress; intellect clear. He confessed he had taken strychnine. The clothing, by his request, had all been removed, as the slightest touch produced a spasm. I administered twenty grains of sulphate of zinc as soon as it could be obtained. This he swallowed with great difficulty, the contact of the solution with his mouth producing trismus and constriction of the throat. The paroxysms came on every three or four minutes. He was conscious of their approach, and entreated us to hold him, to raise him up, or lift him out of bed, till his body became fixed, his head drawn back, and articulation impossible. In this condition of complete opisthotonos, he remained for about a minute, his face livid, and death apparently inevitable. I now resorted to chloroform inhalations, with the happy result of preventing each paroxysm from lasting over a few seconds, or subduing it before the muscles of the back became rigid. So soon as he felt one coming on, I applied the vapor to his mouth; when the muscles were completely relaxed and the breathing natural, I removed it. The convulsions returned regularly till 2 P.M.; the intervals then grew longer until 5, when the paroxysms entirely subsided. For some time after he regained the use of his hands and arms, the legs could not be touched without producing a shock, as if the poles of a battery had been applied. In the eleven hours, he had used over a pound of chloroform. During the night and next day, I found it necessary to relieve the bladder with the catheter. The following evening — thirty-six hours after I first saw him — he was taken home in his carriage, and a week subsequently he walked to my office, although still suffering from soreness and stiffness of the muscles.

In this case, the sulphate of zinc did not produce emesis, nor did I repeat the dose, feeling confident the drug must already have been absorbed. And here I would state that the treatment given in all the books, viz., "give emetics, and persist in their use until free emesis is produced," should at least be modified. If we do not see the patient till a quarter of an hour after the poison is taken, or if convulsions have set in, emesis will surely do much harm. In a case I saw in Philadelphia, in 1868, the patient was nauseated with doses of sulphate of zinc and ipecac. Each attempt at emesis produced the most alarming convulsions. With chloroform to ward off the convulsions till the poison is eliminated from the system, deaths from strychnine will be very rare.—G. W. Copeland, M.D., *in Boston Medical and Surgical Jour.*

---

Abortive Treatment of Furunculi.—According to several observers, as recorded in the French journals, the following method never fails "to take effect:" As soon as there is perceived that characteristic redness, round in form and variable in size, with a culminating point in the center, which, red at first, soon turns to a grayish-white, dip the finger into a little camphorated alcohol, and gently rub the suspected part, especially the middle; moisten the finger, and rub again, in the same manner, eight or ten times, for half a minute each time. After this friction, cover it lightly, with the finger, with camphorated olive oil. It is rare for a blind boil, or furuncle, at the moment of lessening, to resist four applications of this kind. Often they have been seen to dry up and disappear after only one application.—*Boston Medical and Surgical Journal.*

